# Working Memory and the Enactment Effect in Early Alzheimer's Disease

**DOI:** 10.1155/2014/694761

**Published:** 2014-01-28

**Authors:** Lara A. Charlesworth, Richard J. Allen, Suzannah Morson, Wendy K. Burn, Celine Souchay

**Affiliations:** ^1^Institute of Psychological Sciences, University of Leeds, Leeds LS2 9JT, UK; ^2^Towngate House, Towngate Close, Guiseley, West Yorkshire LS20 9PQ, UK; ^3^LEAD UMR CNRS 5022, Pole AAFE, Esplanade Erasme, Universite de Bourgogne, 21065 Dijon, France

## Abstract

This study examines the enactment effect in early Alzheimer's disease using a novel working memory task. Free recall of action-object instruction sequences was measured in individuals with Alzheimer's disease (*n* = 14) and older adult controls (*n* = 15). Instruction sequences were read out loud by the experimenter (verbal-only task) or read by the experimenter and performed by the participants (subject-performed task). In both groups and for all sequence lengths, recall was superior in the subject-performed condition than the verbal-only condition. Individuals with Alzheimer's disease showed a deficit in free recall of recently learned instruction sequences relative to older adult controls, yet both groups show a significant benefit from performing actions themselves at encoding. The subject-performed task shows promise as a tool to improve working memory in early Alzheimer's disease.

## 1. Introduction

Deficits in working memory (WM), a limited capacity system that supports the online manipulation and temporary storage of information [1], are considered to be a hallmark of Alzheimer's disease (AD), even in its earliest stages [2]. Deficits in span tasks [3, 4] and dual task procedures [5, 6] emerge in the early stages of AD, and have been attributed to central executive dysfunction [7]. Of particular interest is the finding that individuals at genetic risk of developing AD show poor WM performance relative to those not at genetic risk [8, 9], highlighting the potential of such tasks to detect early AD. This study considered the usefulness of the subject-performed task (SPT) manipulation [10] in improving WM in early AD and healthy older adult controls, in a task measuring the ability to verbally repeat short sequences of instructions.

The subject-performed task [10] involves verbally presenting participants with words or instructions consisting of sets of simple actions (e.g., “open the book”), which they are required to enact during this encoding phase. Recall of the actions is then subsequently tested, typically via verbal recall or recognition. In general, research shows that enacted encoding facilitates later memory performance, relative to control conditions in which no enactment occurs during encoding [11]. Cohen [10] originally hypothesised that SPT effects are nonstrategic in nature, such that encoding during SPT does not rely on active verbal or organisational strategies that are necessary during basic verbal encoding. The enactment effect may also be attributable to the development of a richer set of representations supporting performance including visual, spatial, and motoric information [12], or specifically the impact of increased motor coding [13]. Alternatively Kormi-Nouri [14] proposes that enacted encoding is strategic in nature and that it is the involvement of the self during SPT that leads to enhanced remembering.

SPT has been found to enhance recall in several clinical groups, including Parkinson's disease [15] and autism spectrum disorder (ASD) [16], yet research with AD participants has generated mixed findings. Several studies have explored this effect, all using episodic long-term memory (LTM) tasks [17–20]. The first study to investigate SPT in individuals with AD failed to find an enactment effect on LTM [17], and of those studies that have observed an effect, the majority have found it in instances of cued recall [18] but not free recall [19]. In contrast, the most recent study to investigate the enactment effect in mild AD patients demonstrated superior recall for SPT relative to verbal-only encoding tasks in free recall, as well as in semantic-cued recall and object-cued recall [20]. In fact, this study demonstrated that the benefit of object-cued recall was greater for AD patients than neurologically intact older adults, thus highlighting encoding specificity as a principle that might enhance recall in AD.

To summarize, the present study examines the advantage of SPT over verbal-only encoding tasks in individuals with early AD using a recently developed WM task. To our knowledge, this is the first study of its type to adopt a WM approach to enactment with AD patients. The present study uses a modified version of the following instruction task [21], which was designed to explore links between WM decrements and difficulty in retaining, repeating, and implementing complex instructions. In the original version of this task, children heard verbal instructions (e.g., “Touch the red pencil and put it in the black box”) and were required to either perform the sequence or repeat it immediately after presentation. As a WM task, it features a minimal delay between presentation and test, and instruction sequences that are comparatively shorter than those most frequently used in the SPT literature. As the instruction task has been shown to rely heavily on WM abilities [21], and because patients with early AD have WM deficits, we predicted that this group would perform poorer relative to an older adult control group. We also predicted that memory performance would vary depending on encoding context. It was expected that healthy older adult control participants would show a substantial benefit of encoding-based enactment [12]. Furthermore, we developed two contrasting sets of predictions for the AD group. As the benefit of SPT has been suggested to rely on the involvement of the self [14], and because research suggests that AD might be accompanied by a disrupted sense of self [22] (but see [23] for evidence of an intact self-reference effect in AD), we might predict that individuals with AD will fail to benefit from enacted encoding. In contrast, if instead the benefit of enacted encoding relies more on automatic, nonstrategic and multimodal encoding, we predict that SPT will enhance remembering in AD patients, as well as older adult controls. In other words, the potential nonstrategic nature of SPT might allow patients to overcome the executive demands involved in constructing memory representations and thus facilitate their recall performance.

## 2. Method

### 2.1. Participants

Twelve individuals diagnosed with mild AD (5 males) and two diagnosed with mild cognitive impairment (MCI; both female) were recruited for participation from a memory clinic in Leeds (UK), and all received formal diagnosis by a psychiatrist. A number of participants were being medicated with acetylcholinesterase inhibitors at the time of testing, but medication was stabilised for at least 8 weeks prior to testing. Regarding AD severity, all patients scored above 19 on the Mini Mental State Examination (MMSE) [24] (mean = 23.42, SD = 3.18), both patients with MCI scored 25.

Fifteen older adult controls (5 males) were recruited from a volunteer panel held by the University of Leeds (UK). Older adult controls reported themselves to be in good physical and mental health; none were taking any medication that is known to affect the central nervous system, and all were living independently at the time of testing. Participants were screened for symptoms of AD using the MMSE, all scored above the cut-off of 26 points (mean = 29.07, SD = 0.70).

There were no significant group differences in age (*t*(27) = −1.79, *P* = 0.09, *d* = −0.66) or predicted full scale IQ (FSIQ; *t*(27) = 1.44, *P* = 0.16,  *d* = 0.54) (National Adult Reading Test (NART)) [25]. Mean FSIQ scores were 116.36 (SD = 10.26) (range 92–128) in the AD group and 121.2 (7.77) (range 103–129) in the older adult control group. Mean age of AD participants was 82.43 (6.14) (range 71–92) and 78.60 (5.41) (range 68–90) in the older adult group.

### 2.2. Materials and Procedure

The method used was a modified version of that used by Wojcik et al. [16]. Action-object pairings were generated by combining 8 actions (thumb, spin, push, drag, flip, tap, lift, and shake) with 15 objects (erasers, rulers, pens, boxes, and folders with red, yellow, and blue versions of each). Actions were designed to be visually distinct and simple to comprehend. Each action was combined with an object to create action-object pairs (e.g., tap the yellow ruler). Instruction sequences were then generated; they contained between three and seven action-object pairs; for instance, *flip the red ruler* (1), *then spin the blue pen* (2), *then shake the yellow box* (3) is an example of a three action-object sequence. Importantly, in order to minimize LTM contributions and focus on WM, there were no meaningful preexisting relationships between any of the actions or objects.

As instruction sequences included up to seven action-object pairs, some colours (e.g., red) and objects (e.g., box) appeared more than once within a single instruction sequence, but no sequence used the same particular object (e.g., red box) twice. Participants attempted 5 sequences at each length, beginning with sequences containing 3 pairs before progressing to the next sequence length. This continued until all sequence lengths had been completed or until the participant was unable to correctly recall any action-object pairs from the instruction sequence.

**Figure 1:**
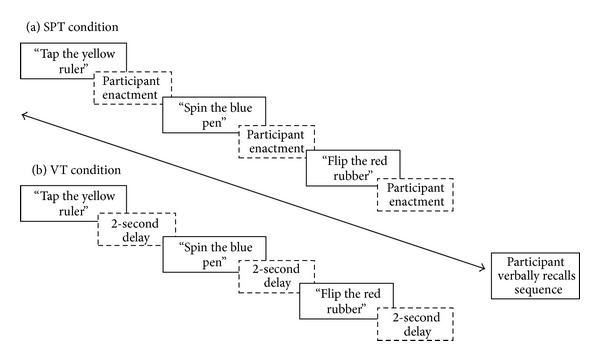
Schematic task diagram of a 3 action sequence. In each condition participants attempted five 3-action sequences, followed by five 4-action sequences, and so on until all sequence lengths were completed or until the participant was unable to correctly recall any action-object pairs from the instruction sequence. SPT: subject-performed task; VT: verbal-only task.

Two encoding conditions were completed by each participant. Instructions were read out loud by the experimenter (verbal-only task, VT) or read by the experimenter and performed by the participant themselves (subject-performed task, SPT) (see Figure 1 for a schematic representation of each encoding task). In the SPT condition, each action-object pair was performed by the participant immediately after verbal presentation; for instance, *“Tap the yellow ruler”* <enactment> *“then spin the blue pen”* <enactment> *“then flip the red rubber”* <enactment>. In the VT condition, participants listened only and were restricted from touching any of the objects. The performance of actions was self-paced in the SPT condition, and a two-second delay separated verbal presentation of each action-object pair in the VT condition to control for this. In both conditions a test phase immediately followed verbal presentation of each instruction sequence, in which participants were asked to verbally recall the entire multiaction sequence. Serial order recall was not explicitly required.

A practice phase, consisting of two practice trials (each involving two action-object pairs), was given prior to each condition, and all actions were demonstrated to participants prior to testing. Instruction sequences and condition order were fully counterbalanced. Conditions were separated by a ten-minute break in which the NART and MMSE were administered to assess cognitive impairment and estimate FSIQ in both groups. Written informed consent was obtained from all participants and full ethical approval was granted by the University of Leeds' ethics committee prior to the start of any testing. Ethical approval for this research was also granted by the NHS ethics committee prior to testing.

## 3. Results

Performance was scored as the mean proportion of elements correctly recalled from each sequence; that is, participants received credit for each individual action, object, or colour correctly recalled. Analysis of serial order recall (elements recalled in the order that they were presented in) yielded evidence of floor effects, with several participants failing to recall any elements in their correct serial positions. Therefore, as serial ordering mechanisms were not of primary interest in this experiment, free order performance (elements recalled regardless of original order) is reported here. Analysis was carried out on three action-object pair sequences and four action-object pair sequences, as all participants in both groups completed these sequence lengths.

**Figure 2:**
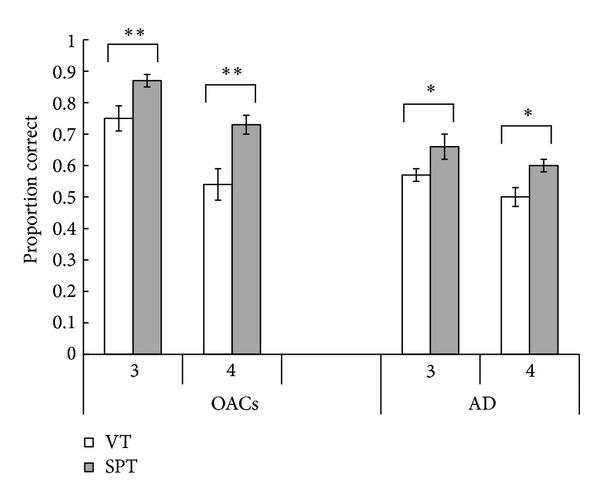
Mean proportion of total elements correctly recalled from each action-object instruction sequence. Error bars represent standard error of the mean. OACs: older adult controls; AD: individuals with Alzheimer's disease; VT: verbal-only task; SPT: subject-performed task; **P = 0.001; *P < 0.01.

Mean performance levels are displayed in Figure 2. A 2 (group) × 2 (encoding condition) × 2 (sequence length) mixed ANOVA was performed. This revealed a main effect of group, *F*(1,27) = 14.16, *P* = 0.001, and *η*_*p*_^2^ = 0.34, such that older adult control participants recalled significantly more elements from the action-object instruction sequences than did AD patients. A main effect of encoding condition was also found, *F*(1, 27) = 46.71, *P* < 0.001, and *η*_*p*_^2^ = 0.63, and this was in the direction predicted, as recall was significantly higher in the SPT condition compared with the VT condition, across group and sequence length. Analysis also revealed a main effect of sequence length, *F*(1,27) = 47.5, *P* < 0.001, and *η*_*p*_^2^ = 0.64, with the proportion of correctly recalled information being higher for 3 action-object pair sequences than for 4 pair sequences.

There was no significant interaction between encoding condition and group, *F*(1,27) = 2.67, *P* = 0.11, and *η*_*p*_^2^ = 0.09, though the effects of SPT were slightly larger in the older adult group. There was also no interaction between condition and sequence length, *F*(1,27) = 1.09, *P* = 0.31, and *η*_*p*_^2^ = 0.04. Overall, SPT led to improved performance across participant groups and number of action-object pairs.

A significant interaction between sequence length and group was found, *F*(1,27) = 10.12, *P* = 0.004, and *η*_*p*_^2^ = 0.27. Inspection of Figure 2 suggests that older adults show a greater proportional decline when sequence length is increased from 3 action-object pair sequences to 4 pair sequences. This is regardless of encoding condition, as there was no significant 3-way interaction, *F*(1,27) = 0.87, *P* = 0.36, and *η*_*p*_^2^ = 0.04. In order to explore the group × length interaction, data was collapsed across encoding condition, and paired samples *t*-tests indicated that increasing sequence length had a greater effect on the recall performance of older adult controls than it did on AD participants, *t*(29) = 7.08, *P* < 0.001, and *d* = 1.38, and *t*(27) = 2.95, *P* = 0.006, and *d* = 0.56, respectively. This is likely due to the already relatively poor performance of AD participants at the shorter sequence length. All analyses were repeated without MCI participants to determine whether this had any effect on findings; this was not the case; all basic patterns of findings were replicated.

## 4. Discussion

This study examined the memory performance of individuals with early AD and older adult controls on an instruction task that required the temporary storage in WM and subsequent recall of action-object sequences, following self-enactment (SPT) or a baseline control condition (VT). Participants with AD tended to show a deficit in remembering relative to older adult controls, supporting extensive existing literature indicating WM deficits in early AD [2]. The primary focus of the present study was to establish whether the WM performance of AD patients would benefit from self-enactment at encoding. The findings reveal that verbal recall in both older adults and individuals with AD was significantly facilitated by the performance of actions on objects at encoding. To our knowledge, this is the first study to indicate a beneficial enactment effect on WM in older adults and AD patients, and it suggests that this manipulation might have useful applications for the amelioration of cognitive deficits in early AD. More generally, the basic task of following and recalling instructions might be useful in detecting early stage AD. The ability to follow instructions has been observed to be particularly deficient in children identified as having poor WM [26]; an analogous deficit may also emerge as a result of AD.

What implications might these findings have both for the enactment effect in WM and for the nature of the cognitive deficit in AD? Wojcik et al. [16] observed substantial benefits of encoding-based enactment in a similar WM task in typical children and children with autism spectrum disorder, though accuracy was measured by physical enactment rather than verbal recall. Taken together, these findings indicate a positive encoding-based enactment effect in WM across populations and response measures. This benefit might reflect a development of a richer set of representations supporting performance including visual, spatial, and motoric information [12] or specifically the impact of increased motor coding [19]. Linked to this, SPT-based enactment has also been attributed to a boost in item-based encoding, possibly at the expense of relational information [27], which may be relevant to the improvements observed in the present study on a task that did not emphasize serial order. If enactment does indeed lead to capture of information from multiple sources, one storage capacity for integrating and retaining such information may be the episodic buffer component of WM recently developed by Baddeley [1].

An important aspect of many theoretical approaches to enactment/SPT is that any gains from this manipulation are automatic and nonstrategic in nature [10]. Alzheimer's disease is characterised by a relative preservation of automatic cognitive processes and a progressive loss of controlled cognitive processes [28]. This may help explain why the AD group were able to also benefit from this manipulation despite their possible deficits in WM control and executive ability [7]. This observation of significant enactments in WM on free recall tasks differs from some findings in the LTM literature [17, 19], though it fits with work by Lekeu and colleagues [20]. A common factor between that work and the present study is the availability of cues at both encoding and retrieval. Whilst previous research by Herlitz et al. [19] found no memory improvement from SPT on free recall tasks, they showed that AD patients experience an enactment effect in semantic-cued recall. In fact, Herlitz and colleagues [19] demonstrated that enacted encoding is sufficient to improve the LTM performance of patients with severe dementia when semantic cues are present at recall. This suggests that patients require support at both encoding and retrieval in order for enhanced recall via self-performance. In our study, objects remained on view in the response phase, though recall was verbal in nature. It may be that the enactment effect is indeed nonstrategic in nature, but in AD it relies on cue availability in order to enable significant performance facilitation. In contrast, Kormi-Nouri [14] has argued that enactment emphasizes involvement of the self, a form of processing that has been suggested to be impaired in AD [22]. The present observation that significant effects of this manipulation were observed in AD might suggest that enactment does not particularly engage the self when used in WM tasks. However, conclusions on this issue are necessarily tentative, and the fact that enactment had a slightly larger benefit for older adults than AD patients (though the interaction was not significant) means further research will be necessary.

## 5. Conclusion

Our results demonstrate a positive encoding-based enactment effect in WM across older adults and patients with early AD. Findings also support previous research which shows that recall is facilitated in AD by the availability of cues at both encoding and retrieval. Due to the nature of the cognitive deficits in AD, replication using this group and extension to similar paradigms might prove useful in elucidating the mechanisms responsible for the enactment effect. Of particular importance is the role that this manipulation might play in ameliorating the cognitive deficits that present in early AD.
